# Imaging genetic association analysis of triple-negative breast cancer based on the integration of prior sample information

**DOI:** 10.3389/fgene.2023.1090847

**Published:** 2023-02-22

**Authors:** Shipeng Ning, Juan Xie, Jianlan Mo, You Pan, Rong Huang, Qinghua Huang, Jifeng Feng

**Affiliations:** ^1^ Department of Breast Surgery, Guangxi Medical University Cancer Hospital, Nanning, China; ^2^ Department of Clinical Laboratory, Guangxi Medical University Cancer Hospital, Nanning, China; ^3^ Department of Anesthesiology, Maternal and Child Health Hospital of Guangxi Zhuang Autonomous Region, Nanning, China

**Keywords:** imaging genetics, triple-negative breast cancer, NMF, prior information, immunity, prognosis, biomarkers

## Abstract

Triple-negative breast cancer (TNBC) is one of the more aggressive subtypes of breast cancer. The prognosis of TNBC patients remains low. Therefore, there is still a need to continue identifying novel biomarkers to improve the prognosis and treatment of TNBC patients. Research in recent years has shown that the effective use and integration of information in genomic data and image data will contribute to the prediction and prognosis of diseases. Considering that imaging genetics can deeply study the influence of microscopic genetic variation on disease phenotype, this paper proposes a sample prior information-induced multidimensional combined non-negative matrix factorization (SPID-MDJNMF) algorithm to integrate the Whole-slide image (WSI), mRNAs expression data, and miRNAs expression data. The algorithm effectively fuses high-dimensional data of three modalities through various constraints. In addition, this paper constructs an undirected graph between samples, uses an adjacency matrix to constrain the similarity, and embeds the clinical stage information of patients in the algorithm so that the algorithm can identify the co-expression patterns of samples with different labels. We performed univariate and multivariate Cox regression analysis on the mRNAs and miRNAs in the screened co-expression modules to construct a TNBC-related prognostic model. Finally, we constructed prognostic models for 2-mRNAs (IL12RB2 and CNIH2) and 2-miRNAs (miR-203a-3p and miR-148b-3p), respectively. The prognostic model can predict the survival time of TNBC patients with high accuracy. In conclusion, our proposed SPID-MDJNMF algorithm can efficiently integrate image and genomic data. Furthermore, we evaluated the prognostic value of mRNAs and miRNAs screened by the SPID-MDJNMF algorithm in TNBC, which may provide promising targets for the prognosis of TNBC patients.

## 1 Introduction

Triple-negative breast cancer (TNBC) is a specific subtype of breast cancer (BC) (Sukumar et al., 2021). Compared with other BC subtypes, TNBC is generally more aggressive, with rapid disease progression and multiple metastatic diseases at an early stage (Lyons, 2019). Therefore, the prognosis of TNBC patients is poor. Currently, the treatment methods for TNBC patients are mostly chemotherapy and surgery, but the recurrence rate after TNBC treatment is high (Hwang et al., 2019). Therefore, it is necessary to mine novel biomarkers related to the prognosis of TNBC patients.

MicroRNAs (miRNAs) can regulate gene expression, cancer development, and metastasis by binding to target messenger RNAs (mRNAs) (Li et al., 2017; Hong et al., 2020). Previous studies have identified many miRNAs involved in BC pathological progressions, such as miR-205, miR-21, and miR-10b. Overexpression of miR-205 can inhibit the metastasis and invasion of tumor cells in BC (Wang et al., 2013). miR-21 and miR-10 b can promote tumor metastasis and cancer cell proliferation in BC by regulating genes such as NOTCH1, TGFBR2, and TGFB1 (Lee and Jiang, 2017). Therefore, identifying key miRNAs and mRNAs in TNBC may provide new therapeutic targets for TNBC patients. In addition to gene-related biomarkers, cancer tissue-related pathological images provide vital information for disease diagnosis and prognosis. Also, integrating mRNA and image data may contribute to more accurate cancer prognosis prediction (Cheng et al., 2017). Sun et al. (2008) Proposed a new method named GPMKL, which effectively predicted the prognosis of BC patients by entirely using the heterogeneous information in genomic data and image data. Wang et al. (2021) Proposed a unified framework named GPDBN to improve the performance of prognosis prediction in BC patients by integrating genomic data and pathological images. The above studies show that fully mining and integrating the information in mRNA expression data and image data can better predict the prognosis of patients.

Imaging genetics is a non-invasive method that correlates genomics and imaging data to discover significant disease-related modules and explain the pathogenesis of the disease. Most of the previous imaging genetics research has been done on Alzheimer’s disease. Due to the characteristics of small samples and high dimensions of imaging genetics, a variety of effective penalty terms are needed to enable the algorithm to perform effective feature selection in high-dimensional data. Lin et al. (2014) Took into account the prior knowledge of the structure within the data. They used structured sparse canonical correlation analysis (SCCA) to correlate SNPs with fMRI signals at the voxel level to identify more risk loci. In order to solve the association analysis research without prior information, Du et al. (2020) Developed a method based on SCCA to fuse the pairwise group LASSO and graph-guided pairwise group LASSO penalty terms. These two penalty terms are in the SCCA model, the structural information in gene and image data can be automatically recovered, respectively. However, SCCA-related algorithms have high algorithm complexity, and it is time-consuming to perform association analysis on high-dimensional data, and there are few studies on cancer imaging genetics. Some scholars extracted features from the tumor contours of CT images of lung cancer patients, compared and analyzed the imaging features with clinical information and gene expression, and found many imaging features with the predictive ability (Aerts et al., 2014).

In recent years, matrix decomposition technology has been widely used in biological multi-omics analysis and has made significant progress. However, few studies have used this type of technology to integrate cancer imaging genetics data and explore the impact of genetic data on imaging phenotypes. Deng et al. proposed a multi-constrained joint non-negative matrix factorization (MCJNMF) algorithm that integrated PET images and DNA methylation data of sarcomas and successfully discovered co-expression modules associated with lung metastasis. Furthermore, they extended the modality to three dimensions by extending the MCJNMF algorithm. They proposed a multidimensional non-negative matrix factorization (MDJNMF) algorithm that integrated pathological images, DNA methylation data, and copy number variation data from sarcoma data. The mechanism of interaction of the three data in sarcoma patients was successfully discovered (Deng et al., 2021).

This paper proposes a sample prior information-driven multidimensional joint non-negative matrix factorization (SPID-MCJNMF) algorithm, which adds a diagnostic information constraint to the basis matrix based on the MDJNMF algorithm. Specifically, we add the clinical stage information of triple-negative breast cancer patients into the algorithm through the Laplace constraint, which is used to make the samples of the same stage closer in the feature space, and the samples of different stages further in the feature space, allow the algorithm to identify expression patterns across samples of different stages. The results show that compared with several other competitive algorithms, the SPID-MCJNMF algorithm has better reconstruction performance and obtains a significant co-expression module with biological significance. Then, to further mine the biomarkers related to the prognosis of TNBC, we performed the prognosis analysis based on the modules screened by the SPID-MCJNMF algorithm and constructed the mRNA and miRNA-related prognosis models, respectively. Prognostic analysis of external datasets further validated the predictive accuracy of the prognostic model. Our study may provide new targets for the treatment and prognosis of TNBC.

## 2 Method

### 2.1 Joint non-negative matrix factorization (JNMF)

Non-negative matrix factorization (NMF) is a robust dimensionality reduction algorithm widely used in bioinformatics to ensure the non-negativity of the original data. Joint non-negative matrix factorization (JNMF) is evolved from NMF, which solves the disadvantage that NMF can only decompose single-modal data, and its objective function is shown in formula (1).

$$\min\overset{n}{\underset{i=1}{\sum }}{∥X_{I}-WH_{I}∥}_{F}^{2}$$

$$s.t.\;W>0,H_{i}>0,i=1,2,3,..n$$ (1)

Among them, n represents the total number of samples. $X_{I}$ represents the feature matrix of different modal data, each row represents a sample, and each column represents a feature of the sample; it is necessary to ensure that the number of rows of $X_{i}\left(i=1,2,3,\ldots \right)$ is consistent (sample one correspondence), the number of columns (number of features) can be different. W is the base matrix obtained after splicing multiple $X_{i}$ and performing non-negative matrix decomposition, and $H_{i}$ is the multiple coefficient matrices obtained by decomposing. In this paper, $X_{I}$ represents the WSI image, $X_{2}$ represents miRNA, and $X_{3}$ represents mRNA. The coefficient matrices obtained after decomposition are $H_{1}$ , $H_{2}$ , and $H_{3}$ , respectively.

### 2.2 Multi-dimensional joint non-negative matrix factorization (MDJNMF)

The MDJNMF algorithm is proposed by Deng et al. Based on the JNMF algorithm, they added orthogonal constraints to the coefficient matrix to prevent redundant features from affecting the results. In addition, they added the absolute value of the Pearson correlation coefficient between WSI image and genetics as prior information to the algorithm, and its objective function is shown in Eq. 2.

$$\begin{array}{c} \Gamma \left(W,H_{1},H_{2},H_{3}\right)=\min\overset{3}{\underset{l=1}{\sum }}{\left|X\right.}_{l}-{\left.WH_{l}\right|}_{F}^{2}+\alpha {\left|H\right.}_{l}H_{l}^{T}-I_{F}^{2}|+{\left.\gamma_{2}|{\left|H\right.}_{l}\right|}_{1}^{2}\\ -\lambda_{1}Tr\left(H_{1}A_{1}H_{2}^{T}\right)-\lambda_{2}Tr\left(H_{1}A_{2}H_{3}^{T}\right)+{\left.\gamma_{1}|W\right|}_{F}^{2} \end{array}$$ (2)

Where I is the identity matrix, α is used to control the orthogonality of $H_{l}$ , and $\lambda_{1}$ 、 $\lambda_{2}$ 、 $\gamma_{1}$ and $\gamma_{2}$ are hyperparameters that control the strength of each regularization constraint, respectively.

### 2.3 Sample prior information driven multiple dimension joint non-negative matrix factorization (SPID-MDJNMF)

To improve the model’s generalization ability and identify markers associated with breast cancer, this subsection introduces a diagnosis-guided penalty term. By treating each sample as a node in an undirected graph, connections between nodes are used to embed clinical information about the patient. In this paper, we embed the clinical stage information of triple-negative breast cancer patients. If any two nodes are selected, and their diagnosis is the same, then there is a connection between them; if the diagnosis is different, there is no connection. Then the adjacency matrix *A* can be obtained, and its element *A*_*ij*_ can be defined as:

$$A_{ij}=\left\{\begin{array}{c} 1,if\;x_{i}\;and\;x_{j}\;are\;from\;the\;same\;group\\ 0,Others\; \end{array}\right.$$ (3)

Where the group represents the stage of the samples, and if the stage of the i−th and j−th samples is the same, the i−th row and j−th column of matrix A takes the value 1. Furthermore, we define the penalty term for diagnosis guidance as shown in Eq. 3.

$$P\left(W\right)=\overset{k}{\underset{i=1}{\sum }}A_{ij}\left(W_{i}-W_{j}\right)$$ (4)

Where $W_{i}$ and $W_{j}$ represent the i−th and j−th columns of the basis matrix W , respectively. k is the dimensionality reduction. *A*_*ij*_ is each element of the matrix *A∈R* ^*n×n*^, which is used to embed the disease course information of the sample. Furthermore, we introduce a degree matrix *D*, which is a pair whose diagonal elements are angle matrix $D_{ii}=\overset{n}{\underset{j=1}{\sum }}A_{ij}$ . Next, this paper further rewrites $P\left(W\right)$ into the following form:

$$P\left(W\right)=Tr\left(W^{T}LW\right)$$ (5)

L represents the Laplace matrix of L . And L=D−A, and then the objective function of the SPID-MCJNMF algorithm is obtained:

$$\begin{array}{c} \Gamma \left(W,H_{1},H_{2},H_{3}\right)=\min\overset{3}{\underset{l=1}{\sum }}{\left|X\right.}_{l}-{\left.WH_{l}\right|}_{F}^{2}+\alpha {\left|H\right.}_{l}H_{l}^{T}-I_{F}^{2}|+{\left.\gamma_{2}|{\left|H\right.}_{l}\right|}_{1}^{2}\\ -\lambda_{1}Tr\left(H_{1}A_{1}H_{2}^{T}\right)-\lambda_{2}Tr\left(H_{1}A_{2}H_{3}^{T}\right)+\beta Tr\left(W^{T}LW\right)+{\left.\gamma_{1}|W\right|}_{F}^{2} \end{array}$$ (6)

Where β is a hyperparameter that controls the strength of the constraints of the sample prior information. Let $\varphi_{ij}$ and $\phi_{ij}^{\prime }$ be $W_{ij}\geq 0$ and ${\left(H_{I}\right)}_{ij}\geq 0$ . The Lagrange multiplier L is expressed as:

$$L\left(W,H_{l}\right)=\Gamma +Tr\left(\Psi W^{T}\right)+\overset{3}{\underset{l=1}{\sum }}Tr\left(\Phi_{,}H_{l}^{T}\right),\Psi =\left[\Phi_{ij}\right],\Phi =\left[\phi_{ij}^{\prime }\right]$$ (7)

Then L takes the partial derivative with respect to W and $H_{l}$ , and Eq. 8 can be obtained.

$$\frac{\partial L}{\partial W}=\overset{3}{\underset{l=1}{\sum }}\left[-2X_{l}H_{l}^{T}+2WH_{l}H_{l}^{T}\right]+2\gamma_{1}W+2\beta WL+\Psi$$

$$\frac{\partial L}{\partial H_{1}}=-2W^{T}X_{1}+2W^{T}WH_{1}+4\alpha H_{1}H_{1}^{T}H_{1}-4\alpha H_{1}-\lambda_{1}H_{2}A_{1}^{T}-\lambda_{2}H_{3}A_{2}^{T}+\gamma_{2}E_{1}+\Phi_{1}$$

$$\frac{\partial L}{\partial H_{2}}=-2W^{T}X_{2}+2W^{T}WH_{2}+4\alpha H_{2}H_{2}^{T}H_{2}-4\alpha H_{2}-\lambda_{1}{H_{1}A}_{1}+\gamma_{2}E_{2}+\Phi_{2}$$

$$\frac{\partial L}{\partial H_{3}}=-2W^{T}X_{3}+2W^{T}WH_{3}+4\alpha H_{3}H_{3}^{T}H_{3}-4\alpha H_{3}-\lambda_{2}H_{1}A_{2}+\gamma_{3}E_{3}+\Phi_{3}$$ (8)

Among them, the elements of $E_{1}$ , $E_{2}$ and $E_{3}$ are all 1. Based on the KKT condition, the equations of $W_{ij}$ and ${\left(H_{l}\right)}_{ij}$ can be obtained:

$$-\overset{3}{\underset{l=1}{\sum }}{\left(X_{l}H_{l}^{T}\right)}_{ij}w_{ij}+\beta WL+{\left[\overset{3}{\underset{l=1}{\sum }}\left(WH_{l}H_{l}^{T}\right)+\gamma_{1}W\right]}_{ij}w_{ij}=0$$

$${\left(-2W^{T}X_{1}-4\alpha H_{1}-\lambda_{1}H_{2}A_{1}^{T}-\lambda_{2}H_{3}A_{2}^{T}\right)}_{ij}h_{ij}^{1}+{\left[2W^{T}WH_{1}+4\alpha H_{1}H_{1}^{T}H_{1}+\gamma_{2}E_{1}\right]}_{ij}h_{ij}^{1}=0$$

$${\left(-2W^{T}X_{2}-4\alpha H_{2}-\lambda_{1}H_{1}A_{1}\right)}_{ij}h_{ij}^{2}+{\left[2W^{T}WH_{2}+4\alpha H_{2}H_{2}^{T}H_{2}+\gamma_{2}E_{2}\right]}_{ij}h_{ij}^{2}=0$$

$${\left(-2W^{T}X_{3}-4\alpha H_{3}-\lambda_{2}H_{1}A_{2}\right)}_{ij}h_{ij}^{3}+{\left[2W^{T}WH_{3}+4\alpha H_{3}H_{3}^{T}H_{3}+\gamma_{2}E_{3}\right]}_{ij}h_{ij}^{3}=0$$ (9)

Finally, the update rules for W and $H_{l}$ can be expressed as Eq. 10.

$$w_{ij}\leftarrow w_{ij}\frac{{\left(X_{1}H_{1}^{T}+X_{2}H_{2}^{T}+X_{3}H_{3}^{T}+\beta WL\right)}_{ij}}{{\left(WH_{1}H_{1}^{T}+WH_{2}H_{2}^{T}+WH_{3}H_{3}^{T}+\gamma_{1}W\right)}_{ij}}$$

$$h_{ij}^{1}\leftarrow h_{ij}^{1}\frac{{\left(W^{T}X_{1}+2aH_{1}+{\frac{\lambda_{1}}{2}H_{2}A}_{1}^{T}+\frac{\lambda_{2}}{2}H_{3}A_{2}^{T}\right)}_{ij}}{{\left(W^{T}WH_{1}+2\alpha H_{1}H_{1}^{T}H_{1}+\frac{\gamma_{2}}{2}E_{l}\right)}_{ij}}$$

$$h_{ij}^{2}\leftarrow h_{ij}^{2}\frac{{\left(W^{T}X_{2}+2aH_{2}+\frac{\lambda_{1}}{2}{H_{1}A}_{1}\right)}_{ij}}{{\left.{\left(W\right.}^{T}WH_{2}+2\alpha H_{2}H_{2}^{T}H_{2}+\frac{\gamma_{2}}{2}E_{2}\right)}_{ij}}$$

$$h_{ij}^{3}\leftarrow h_{ij}^{3}\frac{{\left(W^{T}X_{3}+2aH_{3}+\frac{\lambda_{2}}{2}{H_{1}A}_{2}\right)}_{ij}}{{\left.{\left(W\right.}^{T}WH_{3}+2\alpha H_{3}H_{3}^{T}H_{3}+\frac{\gamma_{3}}{2}E_{3}\right)}_{ij}}$$ (10)

According to the continuously updated W and $H_{l}$ , make it satisfy the convergence rule, that is, the relative error of reaching the set value or reaching the set number of iterations. We initialize W and $H_{l}$ through singular value decomposition, which effectively avoids the randomness of the initialization process of W and $H_{l}$ . Furthermore, for membership confirmation of co-expression modules, this paper is consistent with previous studies (Li et al., 2017; Hong et al., 2020). In addition, we take the reconstruction error as the criterion for the performance of the algorithm, and its expression is as follows.

$$relative\_error=∥X-WH{∥}_{F}^{2}$$ (11)

### 2.4 Functional enrichment analysis

**FIGURE 1:**
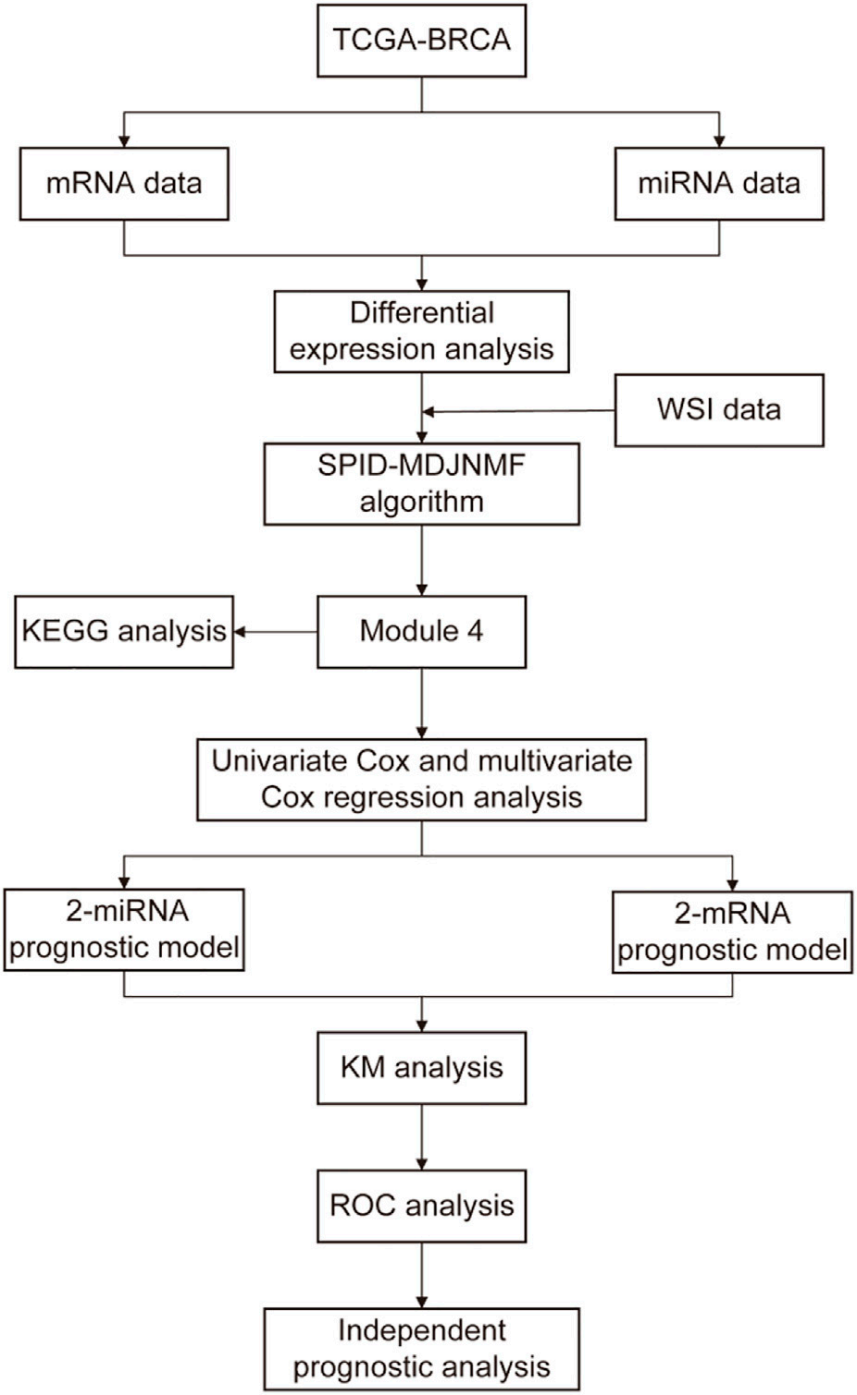
The workflow of this study.

Kyoto Encyclopedia of Genes and Genomes (KEGG) pathway enrichment analyses were used to explore the biological processes in which key mRNAs are involved. In addition, in order to further explore the pathways in which key miRNAs are involved, this paper is based on miRDB, TargetScan, and miRTarBase three databases for key miRNA target gene prediction, and KEGG enrichment analysis for their target genes. Pathways with *p*-values less than 0.05 were considered significant (Figure 1).

### 2.5 Screening and validation of prognosis-related genes

In the prognostic survival analysis, only TNBC patients with a survival time greater than 90 days were retained. Univariate Cox regression analysis was used to identify genes (mRNAs or miRNAs) associated with the prognosis of TNBC patients, and mRNAs or miRNAs with a *p*-value less than 0.05 were reserved as input for multivariate Cox regression analysis. Next, multivariate Cox regression analysis was used to construct mRNA and miRNA-related prognostic models. We then calculated a risk score, which can be used to classify patients in the training cohorts and validation cohorts into high and low-risk groups. The formula for calculating the risk score is as follows:

$$Risk\;Score=\overset{m}{\underset{n=1}{\sum }}coef\left(n\right)*x\left(n\right)$$ (12)

where $coef\left(n\right)$ represent the Cox regression coefficient; x(n) represent the expressive value of each genes, m represents the number of gene. Finally, overall survival (OS) times were compared for the two subgroups in the test and validation datasets by KM analysis to determine the predictive value of the risk model. Receiver operating characteristic (ROC) curves were used to assess the accuracy of risk models by the R package “timeROC.”

## 3 Results

### 3.1 Data source and preprocessing

The data used in this paper are from the TCGA database (https://www.cancer.gov). In this study, the mRNA expression data (mRNA-Seq, 104 cases), miRNA expression data (miRNA-Seq, 102 cases), WSI image data (69 cases), and clinical data (116 cases) of TNBC patients were obtained from the TCGA database. Finally, 69 TNBC samples with mRNA expression data, miRNA expression data, WSI image data, and clinical data were retained. The GEO cohort (https://www.ncbi.nlm.nih.gov/geo/, GSE58812) was used to validate the accuracy of the prognostic model constructed from mRNA expression data. The miRNA data of the TCGA-BRCA cohort (1000 cases) were utilized to validate the accuracy of the prognostic model constructed from the miRNA expression data.

**FIGURE 2:**
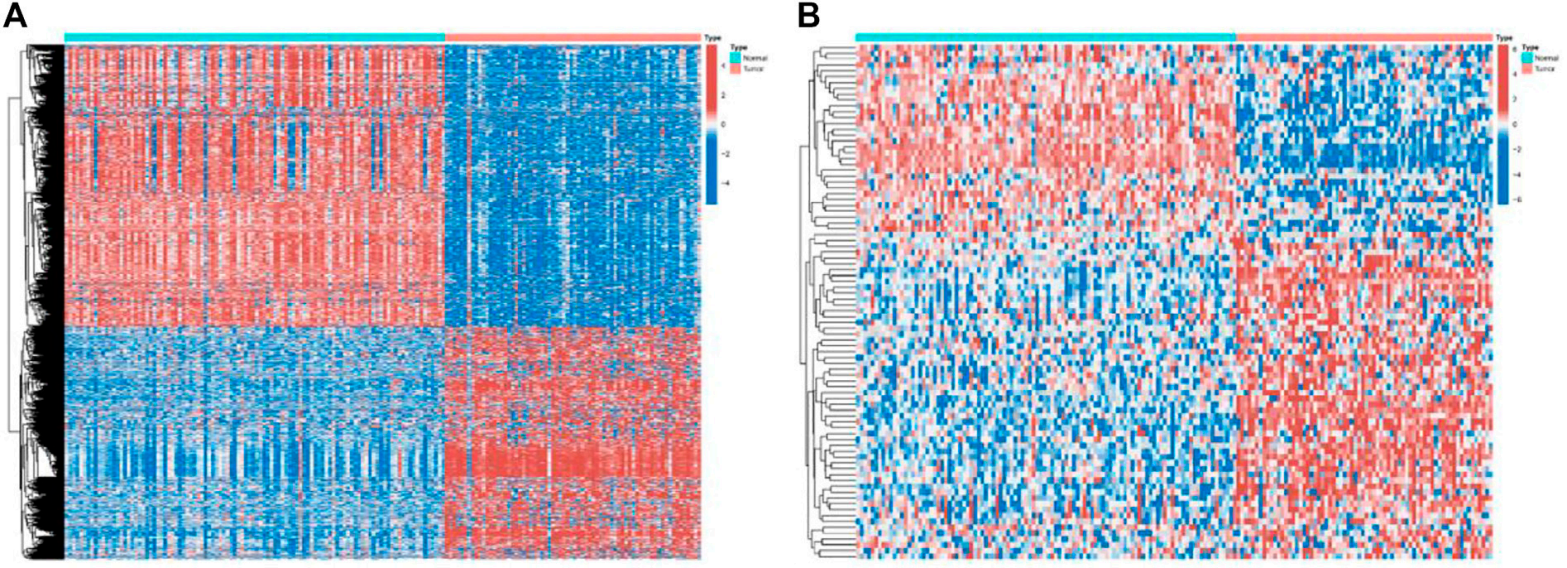
Expressions of the 1438 genes and 524 miRNAs. **(A)** Heatmap of the 1438 genes between the normal (N, blue) and the tumor tissues (T, red). **(B)** Heatmap of the 524 miRNAs between the normal (N, blue) and the tumor tissues (T, red).

In this study, mRNA expression data were differentially analyzed using the “limma” package, and genes with *p*-values less than 0.05 and |logFC|>2 were regarded as differentially expressed genes. Finally, 1438 differentially expressed genes were obtained (Figure 2A), and the expression data of 764 mRNAs were reserved for further analysis through gene annotation. Next, we use the “edgeR” package to normalize the miRNA data (count) by CPM and retain miRNAs with a mean CPM greater than 1. Finally, the expression data of 524 miRNAs were obtained for further analysis (Figure 2B).

Genomic data used in this paper are all from the TCGA database (https://www.cancer.gov). WSI images are from 69 patients with sarcoma. Feature extraction for each WSI image consists of three steps, nuclear segmentation, cell-level feature extraction, and aggregating cell-level features into patient-level features (Phoulady et al., 2016). Based on the experience of previous papers (Cheng et al., 2020), we extracted ten different cell-level features from each segmented nucleus: nuclear area (denoted as area), length of nucleus long and short axes, and long and short axis lengths. The ratio (major, minor, and ratio) of the cell’s mean pixel value in the three channels of RGB (rMean, gMean, and bMean) and the mean, maximum and minimum distances to its neighboring nuclei (distMean, distMax, and distMin) (Cheng et al., 2017). The naming convention for each feature includes cell-level and patient-level features, such as area_bin1, area_mean, etc. In particular, area_bin1 represents the percentage of extremely small cores, and area_bin10 represents the percentage of extremely large cores. Finally, 150 WSI image features are selected as the image data input. We list 150 WSI imaging features in detail in the Supplementary Material. In addition, we provide the difference result files for mRNA and miRNA in Figure 2 (TCGA.diff_mRNA.xls and TCGA. diff_miRNA.xls are in the Supplementary Material).

### 3.2 Hyperparameter selection

**FIGURE 3:**
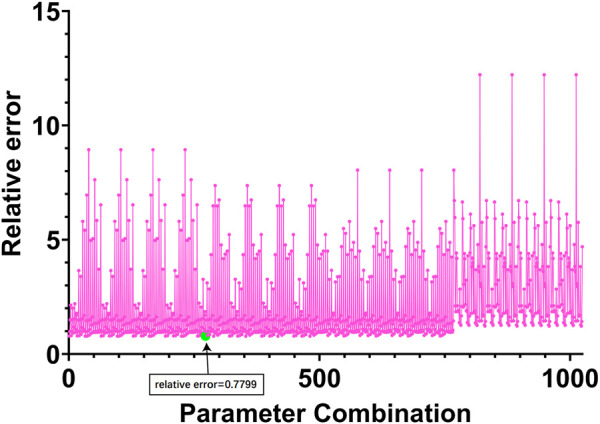
Relative errors corresponding to different parameter combinations. The horizontal coordinate in the figure represents 1024 parameter combinations, and the vertical coordinate represents the index to measure the performance of the algorithm proposed in Eq. 11 in Section 2.3.

The hyperparameter $\lambda_{1}$ , $\lambda_{2}$ , β , $\gamma_{1}$ , $\gamma_{2}$ and the number of co-expression modules K involved in this paper. We conducted experiments on the real dataset, selecting the number of co-expression modules and the remaining four hyperparameters. For the selection of k , since the minimum number of samples/features in the training set is 69, according to the parameter selection experience in the literature (Deng et al., 2020), this paper sets the value of k to 7. For other hyperparameters, we use the grid search method to select parameters. Each parameter is selected from the range of [0.001, 0.01. 0.1. 1], and finally, 1024 parameter combinations are obtained. We take the reconstruction error as the selection basis for selecting all parameters. Furthermore, we use a strategy of early stopping, which stops the iteration when the error no longer decreases, to speed up parameter selection. The hyperparameter selection process is shown in Figure 3 below.

Finally, we selected the 273rd group of parameters corresponding to the smallest relative error, and the relative error of this group of parameters was 0.7799. Among them $\lambda_{1}=0.01$ , $\lambda_{2}=0.001$ , β=0.001 , $\gamma_{1}=0.001$ , $\gamma_{2}=0.01$ .

### 3.3 Selection of co-expression modules

**FIGURE 4:**
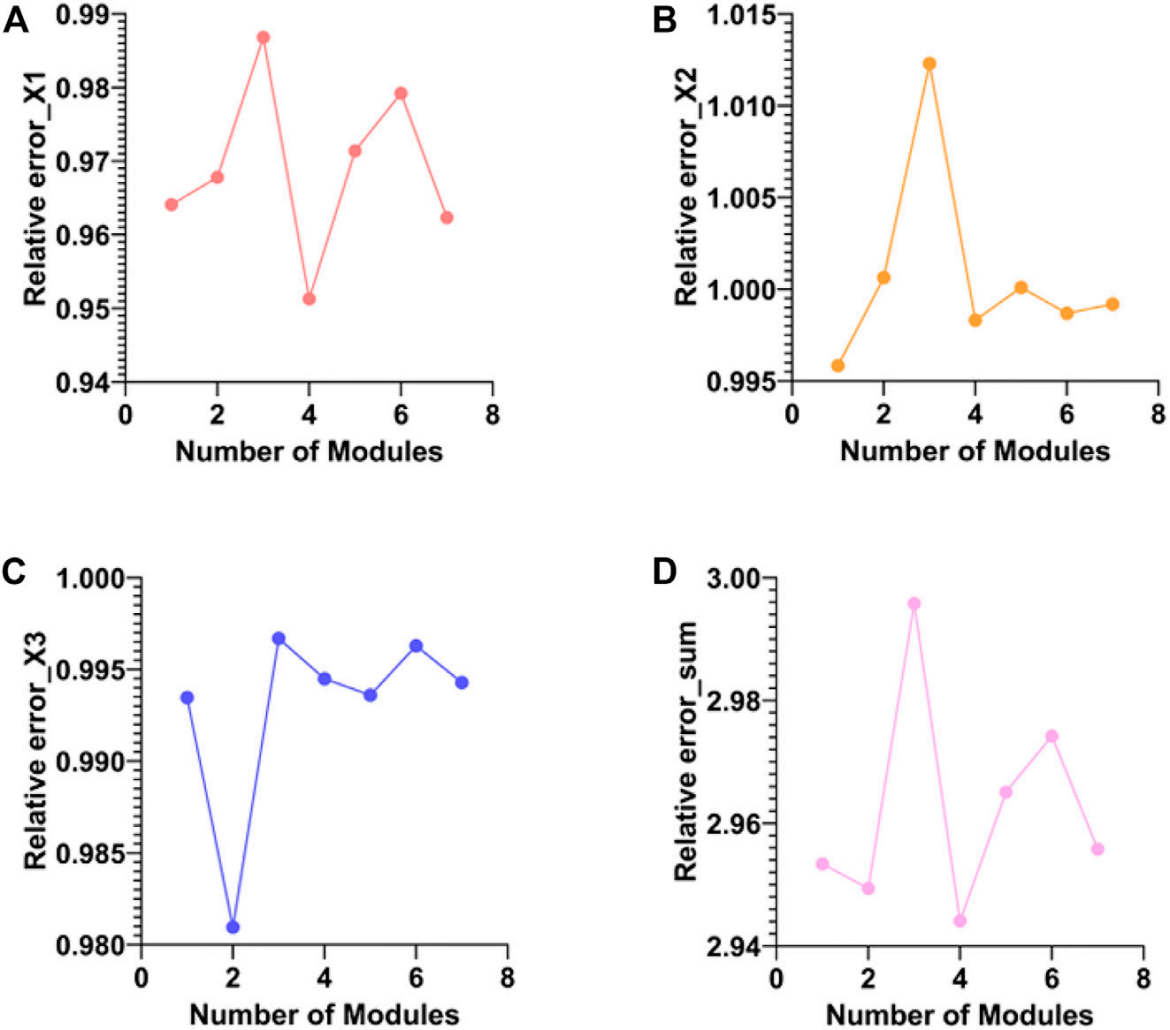
Comparison of Pearson correlation coefficients of three elements in different modules. **(A–C)** are the absolute values of the Pearson correlation coefficients between the original and reconstructed matrices of WSI, miRNA, and mRNA for different modules, respectively. **(D)** is the mean of the absolute value of the Pearson correlation coefficient.

The meaning of the co-expression module is a low-dimensional representation of all the features of the three types of data obtained for the projection of the three types of data into the low-dimensional space. The features of each module equivalent to three types of data with large coefficients in the same projection direction are deposited into the same co-expression module. In the experiments, we obtained a total of 69 co-expression modules. We use the absolute value of the Pearson correlation coefficient between the original matrix and the reconstructed matrix of the three elements in each module as the screening basis. The following figure shows the Pearson correlation coefficient and Pearson correlation of the three elements in all modules and the mean of the three elements (Figures 4A–D).

**FIGURE 5:**
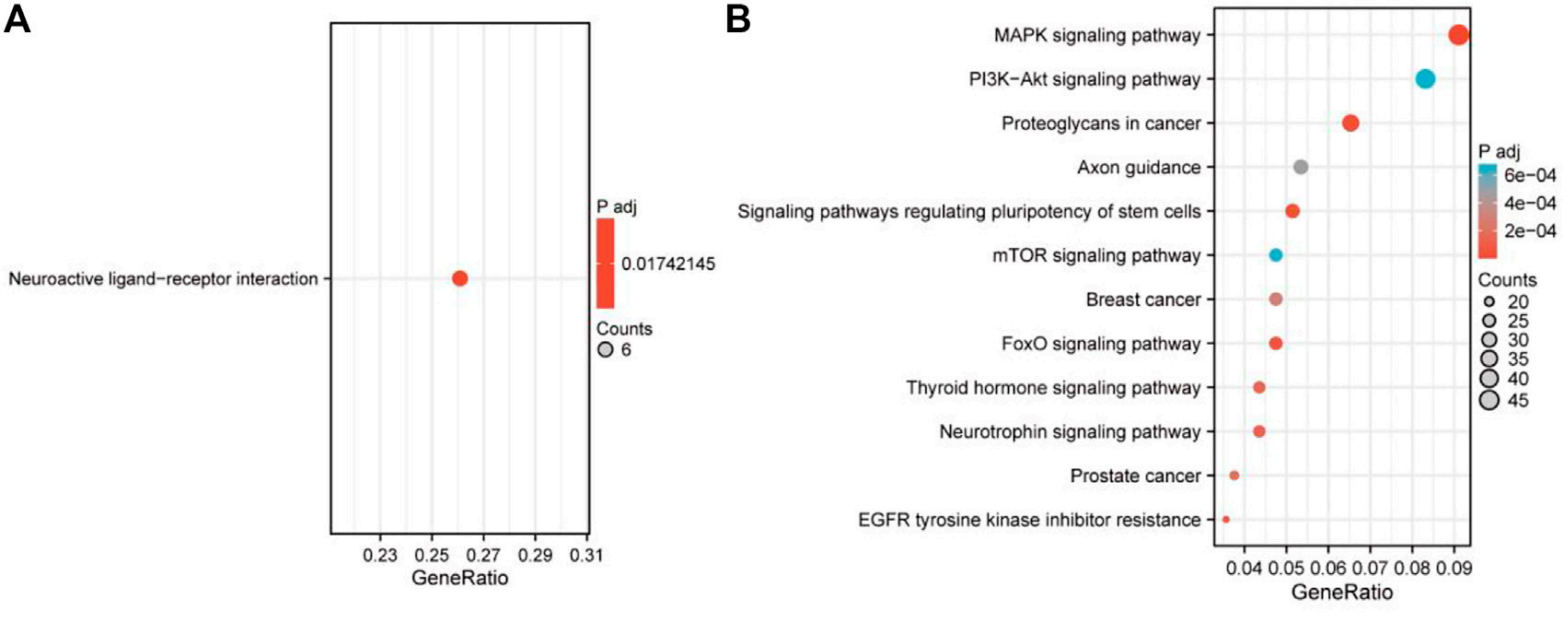
The functional enrichment analysis of module 4 in TNBC. **(A)** The enriched item of the 76 mRNAs. **(B)** The enriched item of the miRNA-related target genes.

As can be seen from Figure 3, module 4 has the smallest total relative error. Therefore, target gene prediction of miRNAs in module 4 was performed in this paper. Next, we performed target gene prediction for the 71 miRNAs in module 4, and the miRNA-mRNA pairs supported by the three miRNAs databases were reserved for further analysis. We predicted 76 target genes. Subsequently, we performed KEGG enrichment analysis on the target genes of mRNAs and miRNAs in module 4 to explore their enriched biological pathways. The results showed that 76 mRNAs in module 4 were enriched in Neuroactive ligand-receptor interaction (Figure 5A). Meanwhile, the target genes of miRNAs in module 4 were mainly enriched in MAPK signaling pathway, Breast cancer, PI3K-Akt signaling pathway, Axon guidance, mTOR signaling pathway, FoxO signaling pathway, and Neurotrophin signaling pathway (Figure 5B). In addition, we provide a list of the identified target genes in the Supplementary Material (miRNA_target_gene.xls).

### 3.4 Comparison with other algorithms

To confirm that the proposed algorithm has good reconstruction performance, we compared the proposed SPID-MDNMF algorithm with the previous JNMF algorithm, the MDJNMF algorithm, under the same experimental conditions. Specifically, the value of k for all three algorithms is 7, and the JNMF algorithm does not have any additional hyperparameters that need to be adjusted. Parameter selection of MDJNMF algorithm is $\lambda_{1}=0.01$ , $\lambda_{2}=0.001$ , $\gamma_{1}=0.001$ , and $\gamma_{2}=0.01$ . Parameter selection of SPID—MDNMF algorithm for $\lambda_{1}=0.01$ , $\lambda_{2}=0.001$ , β=0.001 , $\gamma_{1}=0.001$ , and $\gamma_{2}=0.01$ . The relative error between the original matrix and the reconstructed matrix and the comparison of the Pearson correlation coefficients is shown in Table 1 below.

**TABLE 1:** Comparison of relative errors and correlation coefficients of algorithms.

	$Corr\left(X_{1},WH_{1}\right)$	$Corr\left(X_{2},WH_{2}\right)$	$Corr\left(X_{3},WH_{3}\right)$	relative_error
JNMF	0.8268	0.7710	0.7849	1.1941
MDJNMF	0.9031	0.8529	0.8889	0.8693
SPID-MDJNMF	0.8925	0.8632	0.8870	0.7799

As can be seen from the above table, the proposed SPID-MDJNMF algorithm obtains a minor relative error.

### 3.5 Prognostic biomarkers

**FIGURE 6:**
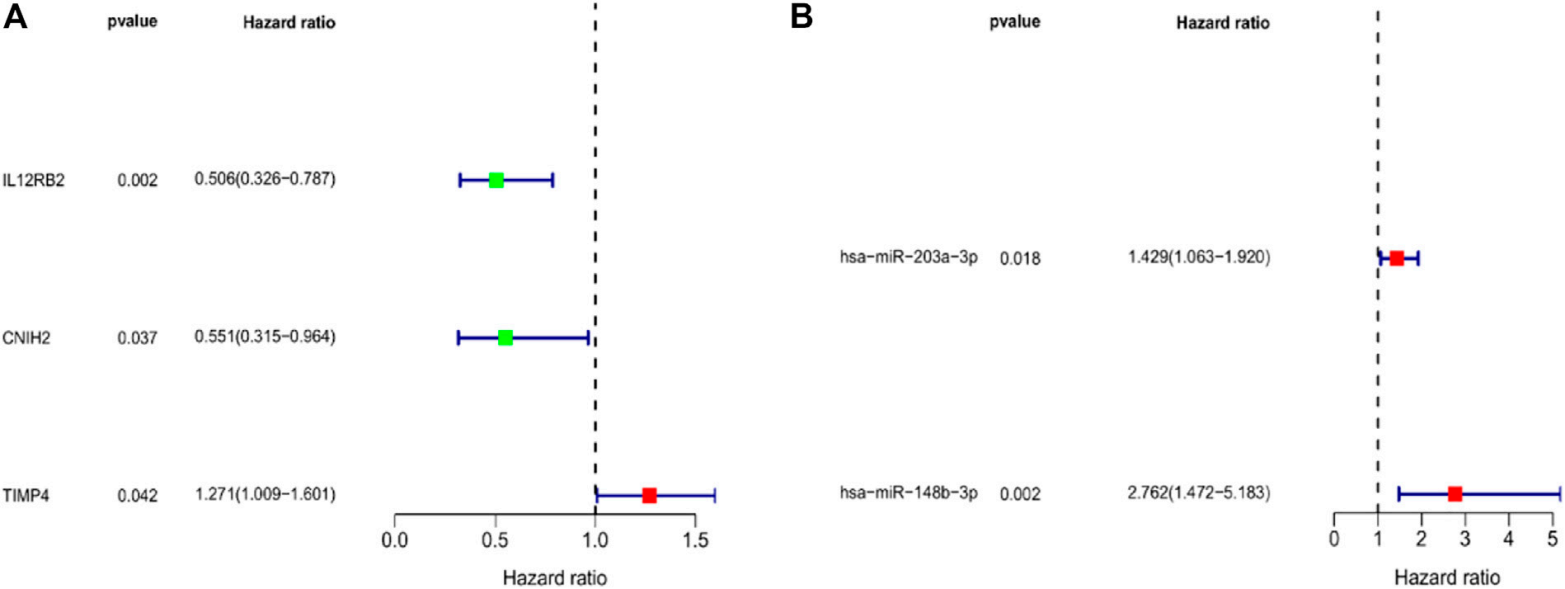
Forest map of univariate regression analyses. **(A)** Univariate Cox regression analysis for identification prognosis-associated mRNAs. **(B)** Univariate Cox regression analysis for identification prognosis-associated miRNAs.

To further screen for key biomarkers, we performed prognostic survival analysis on 76 mRNAs and 71 miRNAs in module 4 to obtain biomarkers that could predict the prognosis of TNBC patients. Univariate Cox regression analysis was performed on the expression data of 76 mRNAs and the expression data of 71 miRNAs, respectively, to screen mRNAs and miRNAs associated with survival time of TNBC patients, mRNAs, and miRNAs with *p*-value <0.05 were retained for further analysis. We obtained a total of 3 mRNAs (IL12RB2, CNIH2 and TIMP4; Figure 6A) and 2 miRNAs (hsa-miR-203a-3p and hsa-miR-148b-3p; Figure 6B) associated with the survival time of TNBC patients (Tables 2, 3). Next, we used multivariate Cox regression analysis (Tables 4, 5) to construct 2-mRNAs-related prognostic models (IL12RB2 and CNIH2) and 2-miRNAs-related prognostic models (hsa-miR -203a-3p and hsa-miR-148b-3p). The risk score of the 2-mRNAs-related prognostic model is expressed as: risk score = (IL12RB2 exp.* −0.60498) + (CNIH2 exp.* −0.43137). The risk score of the 2-miRNAs-related prognostic model is expressed as: risk score = (hsa-miR-203a-3p exp.* 0.403829) + (hsa-miR-148b-3p exp.* 0.997387). TNBC patients in the training dataset (mRNA expression data, TCGA-TNBC) and testing dataset (mRNA expression data, GSE58812) were classified as low-risk group and high-risk group based on the median risk score of the 2-mRNAs-related prognostic model of the TCGA-TNBC cohort. TNBC patients in the training dataset (miRNA expression data, TCGA-TNBC) and the testing dataset (miRNA expression data, TCGA-BRCA) were classified according to the median risk score of the 2-miRNAs-related prognostic model of the TCGA-TNBC cohort into the low-risk group and the high-risk group.

**TABLE 2:** mRNAs associated with TNBC overall survival time were obtained from univariate Cox regression analysis.

Id	HR	HR.95L	HR.95H	P-value
IL12RB2	0.506086	0.325646	0.786507	0.002465
CNIH2	0.550965	0.314765	0.964411	0.036904
TIMP4	1.270984	1.008827	1.601266	0.041899

HR, Hazard ratio; HR.95L, Low 95% CI of HR; HR.95H, High 95% CI of HR.

**TABLE 3:** miRNAs associated with TNBC overall survival time were obtained from univariate Cox regression analysis.

Id	HR	HR.95L	HR.95H	P-value
hsa-miR-203a-3p	1.428542	1.062854	1.920049	0.018078
hsa-miR-148b-3p	2.761921	1.471813	5.182863	0.001559

**TABLE 4:** mRNAs were obtained from multivariate Cox regression analysis.

Id	Coef	HR	HR.95L	HR.95H	P-value
IL12RB2	−0.60498	0.546086	0.350705	0.850315	0.007414
CNIH2	−0.43137	0.649622	0.376577	1.120642	0.121011

Coef, The coefficient of mRNAs (IL12RB2 and CNIH2) correlated with survival; HR, Hazard ratio; HR.95L, Low 95% CI of HR; HR.95H, High 95% CI of HR.

**TABLE 5:** miRNAs were obtained from multivariate Cox regression analysis.

Id	Coef	HR	HR.95L	HR.95H	P-value
hsa-miR-203a-3p	0.403829	1.497548	1.094616	2.048801	0.01156
hsa-miR-148b-3p	0.997387	2.711189	1.509255	4.870316	0.000846

**FIGURE 7:**
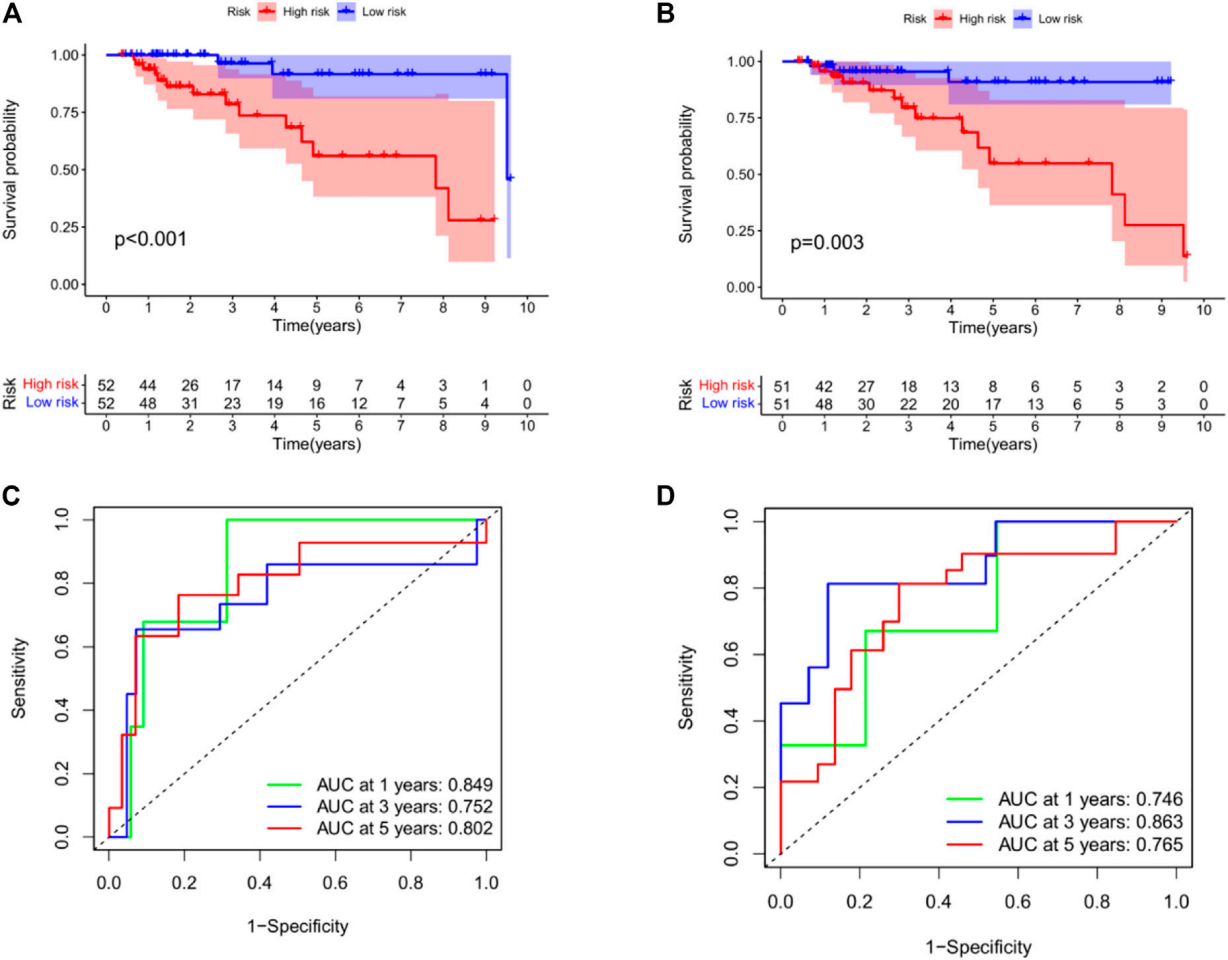
Construction of prognostic model in the training datasets. **(A)** KM curves for the OS of patients in the high- and low-risk groups in the 2 mRNAs-related prognostic model. **(B)** KM curves for the OS of patients in the high- and low-risk groups in the 2 miRNAs-related prognostic model. **(C)** Time-dependent ROC curve analysis of the 2 mRNAs-related prognostic for predicting 1-, 3-, 5-year OS. **(D)** Time-dependent ROC curve analysis of the 2 miRNAs-related prognostic for predicting 1-, 3-, 5-year OS. In addition, we used GSE42568 to validate survival in the high - and low-risk groups (see the Supplementary Figure S1 in Supplementary Material).

Subsequently, we performed KM analysis on mRNA and miRNA-related training datasets. The mRNA-related KM curve showed that the OS rate of high-risk patients in the training dataset was significantly lower than that of low-risk patients over 5 years (Figure 7A, *p* < 0.001). The miRNA-related KM curve showed that the OS rate of high-risk patients in the training dataset was significantly lower than that of low-risk patients over 5 years (Figure 7B, *p* = 0.003). To verify the predictive accuracy of the prognostic model, we plotted the 1-, 3-, and 5-year ROC curves of TNBC patients in the mRNA as well as miRNA-related training datasets. The mRNA-related ROC curve showed that the 2-mRNAs prognostic model we constructed could predict the 1-year (AUC = 0.849), 3-year (AUC = 0.752), and 5-year (AUC = 0.802) survival rates of TNBC patients with high accuracy (Figure 7C). The miRNA-related ROC curve showed that the 2-miRNAs prognostic model we constructed could predict the 1-year (AUC = 0.746), 3-year (AUC = 0.863), and 5-year (AUC = 0.765) survival rates of TNBC patients with high accuracy (Figure 7D).

**FIGURE 8:**
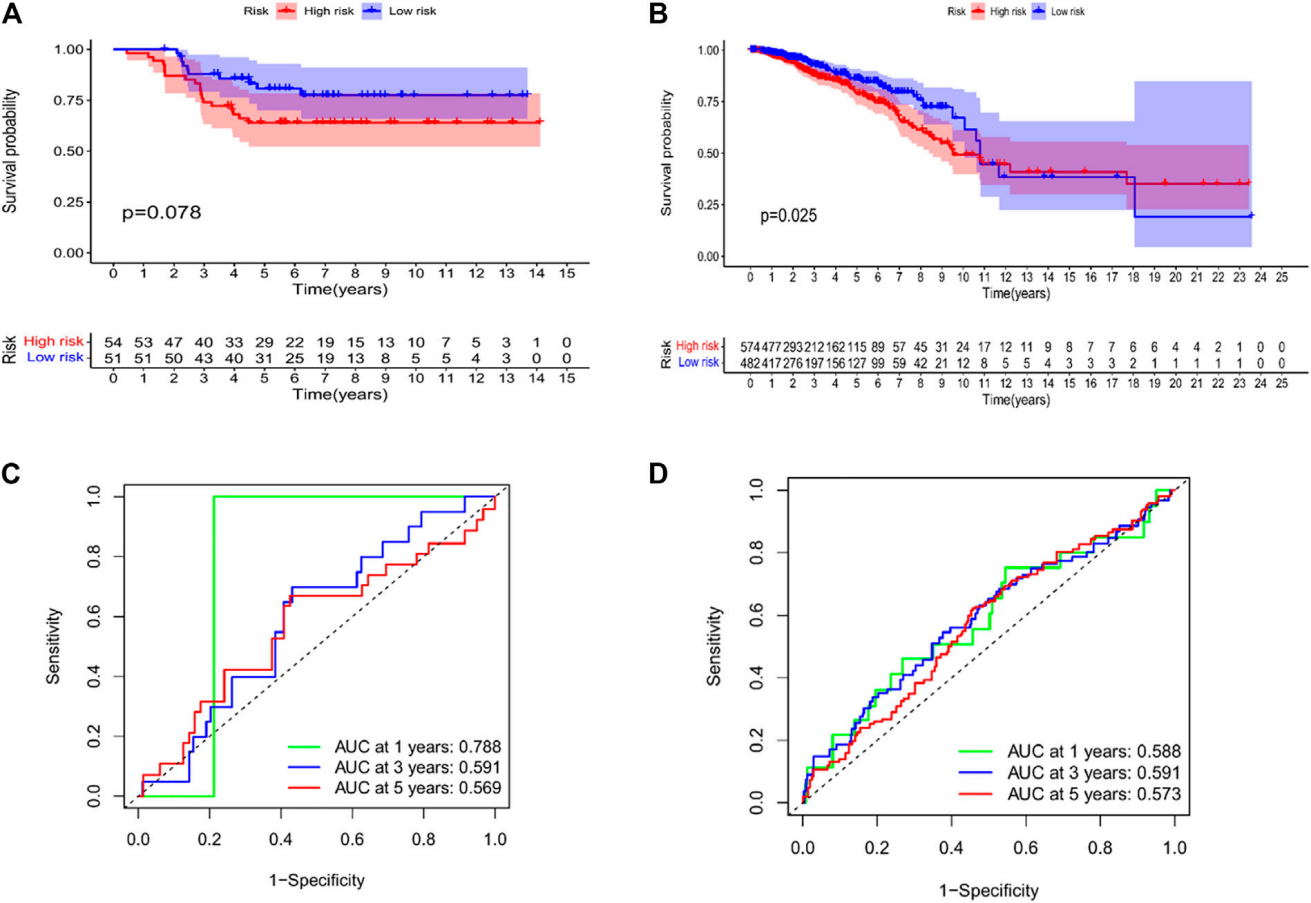
Validation of the risk model in the testing datasets. **(A)** KM curves for the OS of patients in the high- and low-risk groups in the 2 mRNAs-related prognostic model (GSE58812). **(B)** KM curves for the OS of patients in the high- and low-risk groups in the 2 miRNAs-related prognostic model (TCGA-BRCA). **(C)** Time-dependent ROC curve analysis of the 2 mRNAs-related prognostic for predicting 1-, 3-, 5-year OS (GSE58812). **(D)** Time-dependent ROC curve analysis of the 2 miRNAs-related prognostic for predicting 1-, 3-, 5-year OS (TCGA-BRCA).

We performed KM analysis and ROC analysis on the mRNA and miRNA-related test datasets to further verify the predictive accuracy of the constructed prognostic model. The mRNA-related KM curve showed that the OS rate of high-risk patients in the testing dataset was lower than that of low-risk patients over 5 years (Figure 8A, *p* = 0.078). The miRNA-related KM curve showed that the OS rate of high-risk patients in the test dataset was significantly lower than that of low-risk patients over 5 years (Figure 8B, *p* = 0.025). The mRNA correlation ROC curve showed that the 2-mRNAs prognostic model we constructed could predict the 1-year (AUC = 0.788), 3-year (AUC = 0.591), and 5-year (AUC = 0.569) survival of TNBC patients in the test set with certain accuracy rate (Figure 8C). The miRNA-related ROC curve showed that the 2-miRNAs prognostic model we constructed could predict the 1-year (AUC = 0.588), 3-year (AUC = 0.591), and 5-year (AUC = 0.573) survival of TNBC patients in the test set with certain accuracy rate (Figure 8D).

## 4 Discussion

Efficient integration of pathological images and genomic data has been reported to help predict disease prognosis and identify critical targets. To this end, we propose the SPID-MDJNMF algorithm to integrate the pathological image data, mRNAs expression data, and miRNAs expression data of TNBC to identify essential biomarkers in TNBC. Subsequently, we compared the original and reconstructed matrices’ relative errors and Pearson correlation coefficients between the proposed SPID-MDNMF algorithm and the previous JNMF algorithm, the MDJNMF algorithm. The results show that our proposed SPID-MDNMF algorithm has better reconstruction performance. We obtained module 4 through the proposed SPID-MDNMF algorithm. The genomic data and image data in module 4 were left for further analysis.

Next, enrichment analysis was utilized to explore the biological functions of the genes in module 4 in TNBC. The 76 mRNAs in module 4 were enriched in Neuroactive ligand-receptor interaction, Homologous recombination, and Cell cycle. Meanwhile, the target genes of miRNAs in module 4 were mainly enriched in the MAPK signaling pathway, Breast cancer, PI3K-Akt signaling pathway, Axon guidance, FoxO signaling pathway, and Neurotrophin signaling pathway. Some pathways have been shown to be associated with the pathological progression of triple-negative breast cancer, such as BECN1 knockout hinders tumor growth, migration, and invasion by inhibiting the cell cycle and partially inhibiting the epithelial-mesenchymal transition of human triple-negative breast cancer cells (Wu et al., 2018). Alterations in the homologous recombination (HR) system are typical of breast cancer mutant tumors (Belli et al., 2019). Previous studies have shown that the homologous recombination deficiency score may predict the chemotherapeutic range of response to platinum-based neoadjuvant therapy in triple-negative breast cancer (Telli et al., 2016). The RNA-binding protein QKI can inhibit the progression of breast cancer by regulating the RASA1/MAPK signaling pathway (Cao et al., 2021). PIK3CA mutations can confer resistance to chemotherapy in TNBC by activating the PI3K/AKT/mTOR signaling pathway (Hu et al., 2021). The above results suggest that the genes in module 4 may also play an essential role in the occurrence and progression of TNBC.

Subsequently, to screen for biomarkers associated with the prognosis of TNBC patients, we performed univariate and multivariate Cox regression analysis on the 76 mRNAs and 71 miRNAs in module 4. Finally, we constructed a prognostic gene model based on 2 mRNAs (IL12RB2 and CNIH2) and 2 miRNAs (hsa-miR-203a-3p and hsa-miR-148b-3p). Moreover, the mRNA-related and miRNA-related prognostic models we constructed can predict the overall survival of TNBC patients with high accuracy. Epigenetic changes in IL12RB2 play an essential role in the plastic behavior of T Helper 17 (Th17) Cells (Bending et al., 2011). Treg and Th17 cells can influence breast cancer progression through Treg cell-mediated suppression of effector T cell responses (Benevides et al., 2013). Therefore, IL12RB2 may affect breast cancer development by regulating Th17 cells. CNIH2 is an AMPA receptor-binding protein significantly slows AMPAR inactivation (Herring et al., 2013). A previous study found that AMPA antagonists inhibited the proliferation of breast and lung cancer cells *in vitro* (Rzeski et al., 2002). Therefore, CNIH2 may play a role in breast cancer progression through interaction with AMPA. A previous study showed that hsa-miR-203a-3p was upregulated in breast cancer tissues compared with adjacent breast tissues and promoted breast cancer development and carcinogenesis (Cai et al., 2018). Xu et al. found that hsa-miR-203a-3p could inhibit breast cancer progression and metastasis by interacting with circTADA2As (Xu et al., 2019). Breast cancer-related *in vitro* experiments demonstrated that hsa-miR-148b-3p could inhibit tumor cell proliferation and promote breast cancer cell apoptosis by downregulating TRIM59 (Yuan et al., 2019). In breast cancer, miR-148b-3p was found to be associated with disease recurrence and pathological progression by targeting a series of oncogenes (Cimino et al., 2013).

In conclusion, this paper proposes a SPID-MDNMF algorithm that can effectively integrate image data, mRNAs expression data, and miRNAs expression data. Compared with other similar algorithms, the SPID-MDNMF algorithm has better reconstruction performance. Based on module 4 screened by the SPID-MDNMF algorithm, we constructed 2-mRNAs (IL12RB2 and CNIH2) and 2-miRNAs (hsa-miR-203a-3p and hsa-miR, respectively) by performing a prognostic survival analysis on the TCGA-TNBC cohort -148b-3p) prognostic model. The prognostic model can better predict the prognosis of TNBC.

## 5 Conclusion

In summary, we proposed the SPID-MDJNMF algorithm to integrate the imaging genetics data of TNBC patients and obtain the co-expression patterns of TNBC patients in different stages. For the significant co-expressed modules, a variety of bioinformatics analyses were performed to construct a prognostic model for TNBC patients. Multiple genes with prognostic value obtained from screening may be potential biomarkers for TNBC.

## Data Availability

Publicly available datasets were analyzed in this study. This data can be found here: https://www.ncbi.nlm.nih.gov/geo/, GSE58812; TCGA database (https://www.cancer.gov).

## References

[B1] Aerts H. J. W. L. Velazquez E. R. Leijenaar R. T. H. Parmar C. Grossmann P. Carvalho S. (2014). Decoding tumour phenotype by noninvasive imaging using a quantitative radiomics approach. Nat. Commun. 5, 4006. 10.1038/ncomms5006 24892406 PMC4059926

[B2] Belli C. Duso B. A. Ferraro E. Curigliano G. (2019). Homologous recombination deficiency in triple negative breast cancer. Breast 45, 15–21. 10.1016/j.breast.2019.02.007 30818144

[B3] Bending D. Newland S. Krejcí A. Phillips J. M. Bray S. Cooke A. (2011). Epigenetic changes at Il12rb2 and Tbx21 in relation to plasticity behavior of Th17 cells. J. Immunol. 186 (6), 3373–3382. 10.4049/jimmunol.1003216 21307296

[B4] Benevides L. Cardoso C. R. Tiezzi D. G. Marana H. R. Andrade J. M. Silva J. S. (2013). Enrichment of regulatory T cells in invasive breast tumor correlates with the upregulation of IL-17A expression and invasiveness of the tumor. Eur. J. Immunol. 43 (6), 1518–1528. 10.1002/eji.201242951 23529839

[B5] Cai K. T. Feng C. X. Zhao J. C. He R. Q. Ma J. Zhong J. C. (2018). Upregulated miR-203a-3p and its potential molecular mechanism in breast cancer: A study based on bioinformatics analyses and a comprehensive meta-analysis. Mol. Med. Rep. 18 (6), 4994–5008. 10.3892/mmr.2018.9543 30320391 PMC6236224

[B6] Cao Y. Chu C. Li X. Gu S. Zou Q. Jin Y. (2021). RNA-binding protein QKI suppresses breast cancer via RASA1/MAPK signaling pathway. Ann. Transl. Med. 9 (2), 104. 10.21037/atm-20-4859 33569406 PMC7867911

[B7] Cheng J. Han Z. Mehra R. Shao W. Cheng M. Feng Q. (2020). Computational analysis of pathological images enables a better diagnosis of TFE3 Xp11.2 translocation renal cell carcinoma. Nat. Commun. 11, 1778. 10.1038/s41467-020-15671-5 32286325 PMC7156652

[B8] Cheng J. Zhang J. Han Y. Wang X. Ye X. Meng Y. (2017). Integrative analysis of histopathological images and genomic data predicts clear cell renal cell carcinoma prognosis. Cancer Res. 77 (21), e91–e100. 10.1158/0008-5472.CAN-17-0313 29092949 PMC7262576

[B9] Cimino D. De Pittà C. Orso F. Zampini M. Casara S. Penna E. (2013). miR148b is a major coordinator of breast cancer progression in a relapse-associated microRNA signature by targeting ITGA5, ROCK1, PIK3CA, NRAS, and CSF1. FASEB J. 27 (3), 1223–1235. 10.1096/fj.12-214692 23233531

[B10] Deng J. Zeng W. Kong W. Shi Y. Mou X. Guo J. (2020). Multi-constrained joint non-negative matrix factorization with application to imaging genomic study of lung metastasis in soft tissue sarcomas. IEEE Trans. bio-medical Eng. 67 (7), 2110–2118. 10.1109/TBME.2019.2954989 31751222

[B11] Deng J. Zeng W. Luo S. Kong W. Shi Y. Li Y. (2021). Integrating multiple genomic imaging data for the study of lung metastasis in sarcomas using multi-dimensional constrained joint non-negative matrix factorization. Inf. Sci. 576, 24–36. 10.1016/j.ins.2021.06.058

[B12] Du L. Liu K. Yao X. Risacher S. L. Han J. Saykin A. J. (2020). Detecting genetic associations with brain imaging phenotypes in Alzheimer's disease via a novel structured SCCA approach. Med. image Anal. 61, 101656. 10.1016/j.media.2020.101656 32062154 PMC7099577

[B13] Herring B. E. Shi Y. Suh Y. H. Zheng C. Y. Blankenship S. M. Roche K. W. (2013). Cornichon proteins determine the subunit composition of synaptic AMPA receptors. Neuron 77 (6), 1083–1096. 10.1016/j.neuron.2013.01.017 23522044 PMC3652566

[B14] Hong H. C. Chuang C. H. Huang W. C. Weng S. L. Chen C. H. Chang K. H. (2020). A panel of eight microRNAs is a good predictive parameter for triple-negative breast cancer relapse. Theranostics 10 (19), 8771–8789. 10.7150/thno.46142 32754277 PMC7392022

[B15] Hu H. Zhu J. Zhong Y. Geng R. Ji Y. Guan Q. (2021). PIK3CA mutation confers resistance to chemotherapy in triple-negative breast cancer by inhibiting apoptosis and activating the PI3K/AKT/mTOR signaling pathway. Ann. Transl. Med. 9 (5), 410. 10.21037/atm-21-698 33842631 PMC8033310

[B16] Hwang S. Y. Park S. Kwon Y. (2019). Recent therapeutic trends and promising targets in triple negative breast cancer. Pharmacol. Ther. 199, 30–57. 10.1016/j.pharmthera.2019.02.006 30825473

[B17] Lee S. Jiang X. (2017). Modeling miRNA-mRNA interactions that cause phenotypic abnormality in breast cancer patients. PLoS One 12 (8), e0182666. 10.1371/journal.pone.0182666 28793339 PMC5549916

[B18] Li Z. Peng Z. Gu S. Zheng J. Feng D. Qin Q. (2017). Global analysis of miRNA-mRNA interaction network in breast cancer with brain metastasis. Anticancer Res. 37 (8), 4455–4468. 10.21873/anticanres.11841 28739740

[B19] Lin D. Calhoun V. D. Wang Y. P. (2014). Correspondence between fMRI and SNP data by group sparse canonical correlation analysis. Med. image Anal. 18, 6891–6902. 10.1016/j.media.2013.10.010 PMC4007390 24247004

[B20] Lyons T. G. (2019). Targeted therapies for triple-negative breast cancer. Curr. Treat. Options Oncol. 20 (11), 82. 10.1007/s11864-019-0682-x 31754897

[B21] Phoulady H. A. Goldgof D. B. Hall L. O. Mouton P. R. (2016). “Nucleus segmentation in histology images with hierarchical multilevel thresholding,” in International Society for Optics and Photonics.

[B22] Rzeski W. Ikonomidou C. Turski L. (2002). Glutamate antagonists limit tumor growth. Biochem. Pharmacol. 64 (8), 1195–1200. PMID: 12234599. 10.1016/s0006-2952(02)01218-2 12234599

[B23] Sukumar J. Gast K. Quiroga D. Lustberg M. Williams N. (2021). Triple-negative breast cancer: Promising prognostic biomarkers currently in development. Expert Rev. Anticancer Ther. 21 (2), 135–148. 10.1080/14737140.2021.1840984 33198517 PMC8174647

[B24] Sun D. Li A. Tang B. Wang M. (2008). Integrating genomic data and pathological images to effectivepredict breast cancer clinical outcome. Comput. Methods Programs Biomed. 45–53. 10.1016/j.cmpb.2018.04.008 29852967

[B25] Telli M. L. Timms K. M. Reid J. Hennessy B. Mills G. B. Jensen K. C. (2016). Homologous recombination deficiency (HRD) score predicts response to platinum-containing neoadjuvant chemotherapy in patients with triple-negative breast cancer. Clin. Cancer Res. 22 (15), 3764–3773. 10.1158/1078-0432.CCR-15-2477 26957554 PMC6773427

[B26] Wang Z. Li R. Wang M. Li A. (2021). Gpdbn: Deep bilinear network integrating both genomic data and pathological images for breast cancer prognosis prediction. Bioinformatics 37 (18), 2963–2970. 10.1093/bioinformatics/btab185 33734318 PMC8479662

[B27] Wang Z. Liao H. Deng Z. Yang P. Du N. Zhanng Y. (2013). miRNA-205 affects infiltration and metastasis of breast cancer. Biochem. Biophys. Res. Commun. 441 (1), 139–143. 10.1016/j.bbrc.2013.10.025 24129185

[B28] Wu C. L. Zhang S. M. Lin L. Gao S. S. Fu K. F. Liu X. D. (2018). BECN1-knockout impairs tumor growth, migration and invasion by suppressing the cell cycle and partially suppressing the epithelial-mesenchymal transition of human triple-negative breast cancer cells. Int. J. Oncol. 53 (3), 1301–1312. 10.3892/ijo.2018.4472 30015871

[B29] Xu J. Z. Shao C. C. Wang X. J. Zhao X. Chen J. Q. Ouyang Y. X. (2019). circTADA2As suppress breast cancer progression and metastasis via targeting miR-203a-3p/SOCS3 axis. Cell Death Dis. 10 (3), 175. 10.1038/s41419-019-1382-y 30787278 PMC6382814

[B30] Yuan L. Liu Y. Qu Y. Liu L. Li H. (2019). Exosomes derived from MicroRNA-148b-3p-overexpressing human umbilical cord mesenchymal stem cells restrain breast cancer progression. Front. Oncol. 9, 1076. 10.3389/fonc.2019.01076 31696054 PMC6817568

