# Supplementary material for: Imaging genetic association analysis of triple-negative breast cancer based on the integration of prior sample information
Source: Front Genet. 2023 Feb 22;14:1090847. doi: 10.3389/fgene.2023.1090847 (PMC9992804; doi:10.3389/fgene.2023.1090847)
Supplement: Supplementary file 3 [file Table1.DOCX]

# Supplementary Material

## Features of 150 WSI images

area_bin1

area_bin2

area_bin3

area_bin4

area_bin5

area_bin6

area_bin7

area_bin8

area_bin9

area_bin10

area_mean

area_SD

area_skewness

area_kurtosis

area_entropy

major_bin1

major_bin2

major_bin3

major_bin4

major_bin5

major_bin6

major_bin7

major_bin8

major_bin9

major_bin10

major_mean

major_SD

major_skewness

major_kurtosis

major_entropy

minor_bin1

minor_bin2

minor_bin3

minor_bin4

minor_bin5

minor_bin6

minor_bin7

minor_bin8

minor_bin9

minor_bin10

minor_mean

minor_SD

minor_skewness

minor_kurtosis

minor_entropy

ratio_bin1

ratio_bin2

ratio_bin3

ratio_bin4

ratio_bin5

ratio_bin6

ratio_bin7

ratio_bin8

ratio_bin9

ratio_bin10

ratio_mean

ratio_SD

ratio_skewness

ratio_kurtosis

ratio_entropy

rMean_bin1

rMean_bin2

rMean_bin3

rMean_bin4

rMean_bin5

rMean_bin6

rMean_bin7

rMean_bin8

rMean_bin9

rMean_bin10

rMean_mean

rMean_SD

rMean_skewness

rMean_kurtosis

rMean_entropy

gMean_bin1

gMean_bin2

gMean_bin3

gMean_bin4

gMean_bin5

gMean_bin6

gMean_bin7

gMean_bin8

gMean_bin9

gMean_bin10

gMean_mean

gMean_SD

gMean_skewness

gMean_kurtosis

gMean_entropy

bMean_bin1

bMean_bin2

bMean_bin3

bMean_bin4

bMean_bin5

bMean_bin6

bMean_bin7

bMean_bin8

bMean_bin9

bMean_bin10

bMean_mean

bMean_SD

bMean_skewness

bMean_kurtosis

bMean_entropy

distMean_bin1

distMean_bin2

distMean_bin3

distMean_bin4

distMean_bin5

distMean_bin6

distMean_bin7

distMean_bin8

distMean_bin9

distMean_bin10

distMean_mean

distMean_SD

distMean_skewness

distMean_kurtosis

distMean_entropy

distMax_bin1

distMax_bin2

distMax_bin3

distMax_bin4

distMax_bin5

distMax_bin6

distMax_bin7

distMax_bin8

distMax_bin9

distMax_bin10

distMax_mean

distMax_SD

distMax_skewness

distMax_kurtosis

distMax_entropy

distMin_bin1

distMin_bin2

distMin_bin3

distMin_bin4

distMin_bin5

distMin_bin6

distMin_bin7

distMin_bin8

distMin_bin9

distMin_bin10

distMin_mean

distMin_SD

distMin_skewness

distMin_kurtosis

distMin_entropy

## Survival validation of high and low risk groups


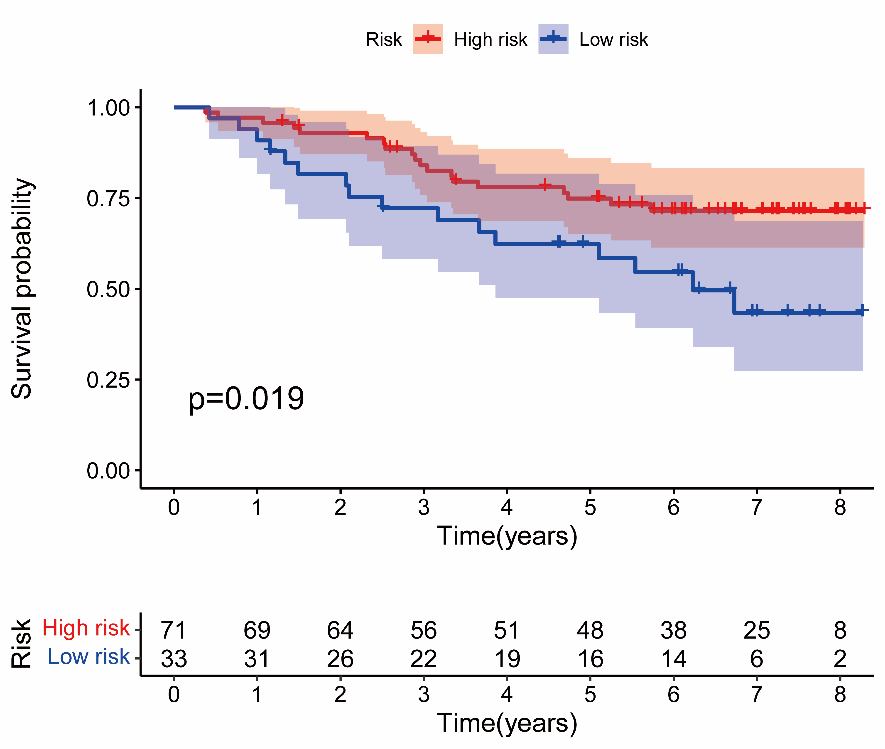


Fig. S1 KM analysis of GSE42568 dataset.
