# Supplementary figures and images for: Imaging genetic association analysis of triple-negative breast cancer based on the integration of prior sample information
Source: Front Genet. 2023 Feb 22;14:1090847. doi: 10.3389/fgene.2023.1090847 (PMC9992804; doi:10.3389/fgene.2023.1090847)

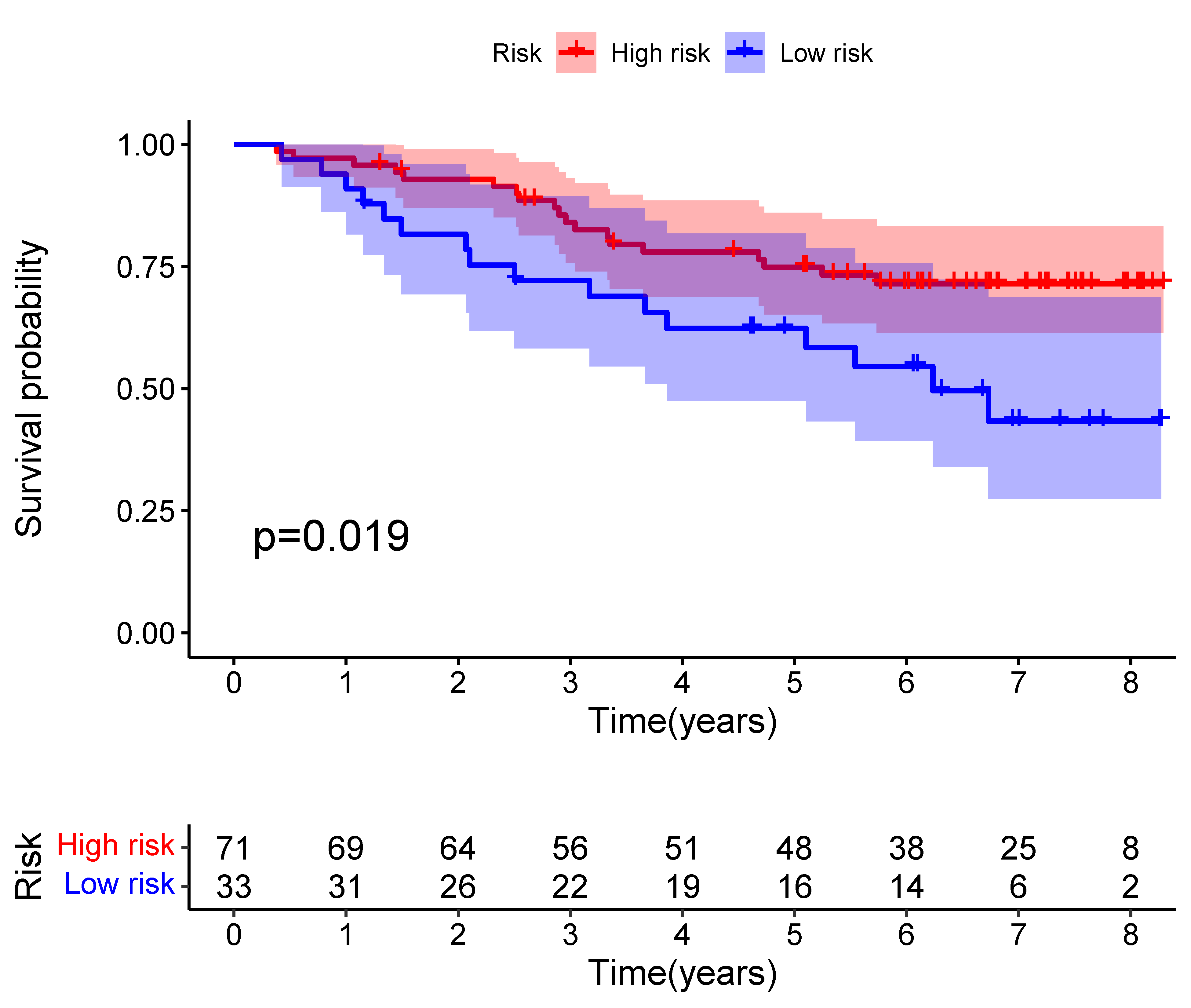

Supplement: Supplementary file 5 [file Image1.TIF]
